# Chlorido{*N*-[(*E*)-2-(diphenyl­phosphan­yl)benzyl­idene]-2-(thio­phen-2-yl)ethan­amine-κ*P*}gold(I)

**DOI:** 10.1107/S1600536811052536

**Published:** 2011-12-14

**Authors:** Haleden Chiririwa, Alfred Muller

**Affiliations:** a Research Center for Synthesis and Catalysis, Department of Chemistry, University of Johannesburg (APK Campus), PO Box 524, Auckland Park, Johannesburg 2006, South Africa

## Abstract

The title compound, [AuCl(C_25_H_22_NPS)], crystallizes with two independent mol­ecules in the asymmetric unit in which the thio­phene fragments are disordered over two sets of sites with 0.537 (10):0.463 (10) and 0.701 (9):0.299 (9) occupancy ratios. In both cases, the thio­phene ring is rotated by approximately 180° for the second component. Important geometrical parameters include Au—P = 2.235 (2) and 2.237 (2) Å, Au—Cl = 2.286 (2) and 2.292 (2) Å, and P—Au—Cl = 177.39 (8) and 172.63 (7)°. Weak inter­molecular C—H⋯Cl inter­actions are observed in the crystal structure.

## Related literature

For general background to the title compound, see: Shaw (1999 ▶); Barnard *et al.* (2004 ▶); Nomiya *et al.* (2003 ▶). For details on the conformational fit of the two mol­ecules using *Mercury*, see: Macrae *et al.* (2006 ▶); Weng *et al.* (2008*a* ▶,*b* ▶).
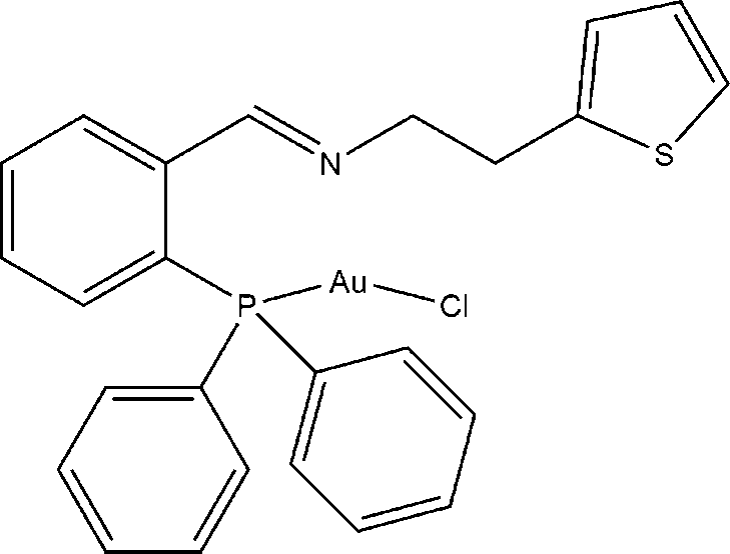


## Experimental

### 

#### Crystal data

[AuCl(C_25_H_22_NPS)]
*M* *_r_* = 631.88
Monoclinic, 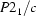

*a* = 11.866 (2) Å
*b* = 10.625 (2) Å
*c* = 37.811 (7) Å
β = 105.63 (3)°
*V* = 4590.8 (16) Å^3^
*Z* = 8
Mo *K*α radiation
μ = 6.70 mm^−1^
*T* = 173 K
0.14 × 0.13 × 0.06 mm

#### Data collection

Bruker APEX DUO 4K CCD diffractometer
Absorption correction: multi-scan (*SADABS*; Bruker, 2007 ▶) *T*_min_ = 0.454, *T*_max_ = 0.689
107056 measured reflections
11023 independent reflections
7856 reflections with *I* > 2σ(*I*)
*R*_int_ = 0.126

#### Refinement

*R*[*F* ^2^ > 2σ(*F* ^2^)] = 0.050
*wR*(*F* ^2^) = 0.109
*S* = 1.12
11023 reflections
642 parameters
238 restraints
H-atom parameters constrained
Δρ_max_ = 1.63 e Å^−3^
Δρ_min_ = −1.24 e Å^−3^

### 

Data collection: *APEX2* (Bruker, 2007 ▶); cell refinement: *SAINT* (Bruker, 2007 ▶); data reduction: *SAINT* and *XPREP* (Bruker, 2007 ▶); program(s) used to solve structure: *SIR97* (Altomare *et al.*, 1999 ▶); program(s) used to refine structure: *SHELXL97* (Sheldrick, 2008 ▶); molecular graphics: *DIAMOND* (Brandenburg & Putz, 2005 ▶); software used to prepare material for publication: *WinGX* (Farrugia, 1999 ▶).

## Supplementary Material

Crystal structure: contains datablock(s) global, I. DOI: 10.1107/S1600536811052536/zq2145sup1.cif

Structure factors: contains datablock(s) I. DOI: 10.1107/S1600536811052536/zq2145Isup2.hkl

Additional supplementary materials:  crystallographic information; 3D view; checkCIF report

## Figures and Tables

**Table 1:** Hydrogen-bond geometry (Å, °)

*D*—H⋯*A*	*D*—H	H⋯*A*	*D*⋯*A*	*D*—H⋯*A*
C28—H28⋯Cl1^i^	0.95	2.81	3.454 (9)	126

Symmetry code: (i) 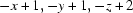.

**Fig. 1.**
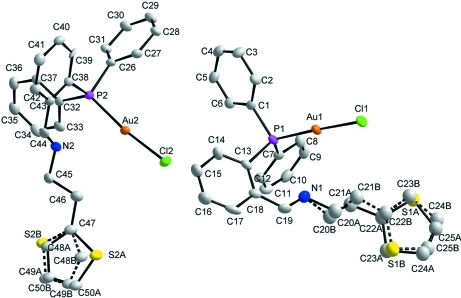
View of the asymmetric unit of the title compound. Displacement ellipsoids are drawn at the 30% probability level. For clarity: a) hydrogen atoms are omitted, b) bonds in part B of the disordered parts are indicated with dotted lines.

**Fig. 2.**
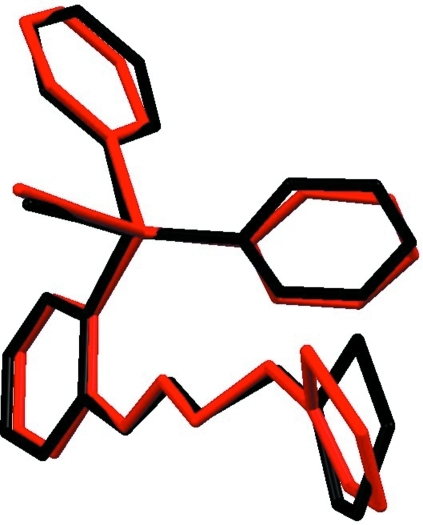
Conformational similarity of the two independent molecules in the asymmetric unit. Hydrogen atoms and the minor components of each disorder have been omitted for clarity.

## supplementary crystallographic information

### Comment

There is a growing interest in the coordination chemistry of iminophosphine ligands containing both hard (N donor) and soft (P donor) Lewis acids. Studies of gold(I) complexes have been related to anti-arthritic (Shaw, 1999), anti-tumor (Barnard *et al.*, 2004) and antimicrobial physiological activities (Nomiya *et al.*, 2003).

The asymmetric unit consists of two crystallographically independent molecules of the title compound, each having distinct features such as P1—Au1—Cl1 = 177.38 (9) and P2—Au2—Cl2 = 172.62 (8)°, respectively (Fig. 1). Bond lengths in the metal coordination environment are comparable and listed in the supplementary material for comparison. The thiophene fragments of each unit also show different packing behavior and had to be treated differently with independent disorder refinements [0.463 (10):0.537 (10) and 0.299 (9):0.701 (9) occupancy ratios]. In both cases the thiophene ring rotated approximately 180° for the second component. Conformational differences between the two independent molecules are highlighted in Figure 2 using Mercury (Macrae *et al.*, 2006; Weng *et al.*, 2008*a*; Weng *et al.*, 2008*b*), with the root mean squared deviation (RMSD) calculated as 0.3274 Å. Weak intermolecular C—H···Cl interactions are observed in the crystal structure (Table 2).

### Experimental

To a dry CH_2_Cl_2_ (10 ml) solution of the precursor [Au(tht)Cl] (tht = tetrahydrothiophene) was added an equimolar amount of *N*-{(*E*)-[2-(diphenylphosphanyl)phenyl]methylidene}-2-thiophen-2-ylethanamine in CH_2_Cl_2_ (10 ml), and stirred at room temperature for 2 hrs. The solvent was reduced and the complex precipitated out on addition of hexane, filtered off, washed with Et_2_O (2 × 5 ml) and dried under vacuum for 4 hrs affording a white precipitate in 55% yield. Crystals suitable for X-ray structure determination were obtained by recrystallization from a CH_2_Cl_2_-hexane mixture at room temperature.

### Refinement

All H atoms were positioned in geometrically idealized positions with C—H = 0.99 Å and 0.95 Å for methylene and aromatic H atoms, respectively. All H atoms were allowed to ride on their parent atoms with *U*_iso_(H) = 1.2*U*_eq_(C). Disorder refinement models for two sites were applied to the thiophenes of each independent molecule in the asymmetric unit (hereafter referred to as molecule 1 and 2 for the unit containing Au1 and Au2, respectively). For the first molecule the disorder was more severe and C20/21 attached to the thiophene also had to be split. Geometrical (*FLAT*) restraints were applied to keep the rings C22A/B—C23A/B—C24A/B—C25A/B—S1A/B and C47A/B—C48A/B—C49A/B—C50A/B—S2A/B planar. Bond distance (*DFIX*) and 1,3 distance similarity restraints (SADI) were applied to obtain reasonable geometries. Ellipsoid displacement (*SIMU* and *DELU*) restraints were also applied to the disordered moieties. Free variables were connected to each disordered component to add to unity, respectively. Occupation parameters of the disordered atoms refined 0.537 (10) and 0.701 (9) for the major components of molecule 1 and 2, respectively. All the above restraints were applied with the default standard deviations for molecule 1. In the case of molecule 2 ellipsoid displacement restraints had to be adjusted to 0.02 and 0.005 for *SIMU* and *DELU*, respectively. Several reflections were omitted (as suggested by the checkCIF procedure) during refinement and can be found from the attached instruction file. The highest residual electron density of 1.63 e.Å^-3^ is 0.92 Å from Au2, representing no physical meaning.

### Crystal data

**Table:**

[AuCl(C_25_H_22_NPS)]	*F*(000) = 2448
*M**_r_* = 631.88	*D*_x_ = 1.828 Mg m^−^^3^
Monoclinic, *P*2_1_/*c*	Mo *K*α radiation, λ = 0.71073 Å
Hall symbol: -P 2ybc	Cell parameters from 12327 reflections
*a* = 11.866 (2) Å	θ = 1.8–29.1°
*b* = 10.625 (2) Å	µ = 6.70 mm^−^^1^
*c* = 37.811 (7) Å	*T* = 173 K
β = 105.63 (3)°	Plate, yellow
*V* = 4590.8 (16) Å^3^	0.14 × 0.13 × 0.06 mm
*Z* = 8	

### Data collection

**Table:**

Bruker APEX DUO 4K CCD diffractometer	11023 independent reflections
graphite	7856 reflections with *I* > 2σ(*I*)
Detector resolution: 8.4 pixels mm^-1^	*R*_int_ = 0.126
ω scans	θ_max_ = 28°, θ_min_ = 1.1°
Absorption correction: multi-scan (*SADABS*; Bruker, 2007)	*h* = −15→15
*T*_min_ = 0.454, *T*_max_ = 0.689	*k* = −14→14
107056 measured reflections	*l* = −49→49

### Refinement

**Table:**

Refinement on *F*^2^	Primary atom site location: structure-invariant direct methods
Least-squares matrix: full	Secondary atom site location: difference Fourier map
*R*[*F*^2^ > 2σ(*F*^2^)] = 0.050	Hydrogen site location: inferred from neighbouring sites
*wR*(*F*^2^) = 0.109	H-atom parameters constrained
*S* = 1.12	*w* = 1/[σ^2^(*F*_o_^2^) + (0.0302*P*)^2^ + 27.231*P*] where *P* = (*F*_o_^2^ + 2*F*_c_^2^)/3
11023 reflections	(Δ/σ)_max_ = 0.003
642 parameters	Δρ_max_ = 1.63 e Å^−^^3^
238 restraints	Δρ_min_ = −1.24 e Å^−^^3^

### Special details

**Table:**

Experimental. The intensity data was collected on a Bruker Apex DUO 4 K CCD diffractometer using an exposure time of 20 s/frame. A total of 1315 frames were collected with a frame width of 0.5° covering up to θ = 28.0° with 99.8% completeness accomplished.
Geometry. All e.s.d.'s (except the e.s.d. in the dihedral angle between two l.s. planes) are estimated using the full covariance matrix. The cell e.s.d.'s are taken into account individually in the estimation of e.s.d.'s in distances, angles and torsion angles; correlations between e.s.d.'s in cell parameters are only used when they are defined by crystal symmetry. An approximate (isotropic) treatment of cell e.s.d.'s is used for estimating e.s.d.'s involving l.s. planes.
Refinement. Refinement of *F*^2^ against ALL reflections. The weighted *R*-factor *wR* and goodness of fit *S* are based on *F*^2^, conventional *R*-factors *R* are based on *F*, with *F* set to zero for negative *F*^2^. The threshold expression of *F*^2^ > σ(*F*^2^) is used only for calculating *R*-factors(gt) *etc*. and is not relevant to the choice of reflections for refinement. *R*-factors based on *F*^2^ are statistically about twice as large as those based on *F*, and *R*- factors based on ALL data will be even larger.

### Fractional atomic coordinates and isotropic or equivalent isotropic displacement parameters (Å^2^)

**Table:**

	*x*	*y*	*z*	*U*_iso_*/*U*_eq_	Occ. (<1)
Au1	0.23553 (3)	0.21978 (3)	1.006902 (8)	0.03632 (9)	
Cl1	0.23618 (19)	0.1881 (2)	1.06676 (5)	0.0505 (5)	
N1	0.0965 (7)	0.0238 (8)	0.9537 (2)	0.056 (2)	
P1	0.23365 (16)	0.2413 (2)	0.94794 (5)	0.0337 (4)	
C1	0.3179 (6)	0.3787 (7)	0.9410 (2)	0.0326 (17)	
C2	0.3387 (7)	0.4763 (8)	0.9659 (2)	0.047 (2)	
H2	0.3074	0.4728	0.9865	0.056*	
C3	0.4050 (8)	0.5803 (8)	0.9612 (3)	0.053 (2)	
H3	0.418	0.6472	0.9785	0.063*	
C4	0.4507 (8)	0.5860 (8)	0.9322 (3)	0.053 (2)	
H4	0.4972	0.6561	0.9293	0.063*	
C5	0.4292 (7)	0.4879 (8)	0.9064 (3)	0.049 (2)	
H5	0.4596	0.4927	0.8856	0.059*	
C6	0.3654 (7)	0.3865 (8)	0.9108 (2)	0.0411 (19)	
H6	0.3526	0.32	0.8934	0.049*	
C7	0.3049 (6)	0.1130 (7)	0.9301 (2)	0.0360 (18)	
C8	0.4113 (7)	0.0707 (8)	0.9517 (2)	0.045 (2)	
H8	0.444	0.106	0.9753	0.053*	
C9	0.4705 (8)	−0.0243 (9)	0.9385 (3)	0.054 (2)	
H9	0.5442	−0.0531	0.953	0.065*	
C10	0.4221 (9)	−0.0764 (9)	0.9044 (3)	0.056 (2)	
H10	0.4621	−0.1418	0.8956	0.067*	
C11	0.3173 (9)	−0.0345 (9)	0.8833 (3)	0.056 (2)	
H11	0.2833	−0.0712	0.8599	0.067*	
C12	0.2605 (7)	0.0620 (8)	0.8960 (2)	0.043 (2)	
H12	0.189	0.0934	0.8808	0.052*	
C13	0.0879 (6)	0.2660 (8)	0.9165 (2)	0.0374 (18)	
C14	0.0683 (7)	0.3772 (9)	0.8960 (2)	0.049 (2)	
H14	0.1316	0.4334	0.8973	0.059*	
C15	−0.0429 (7)	0.4076 (10)	0.8735 (2)	0.056 (2)	
H15	−0.0537	0.4827	0.8594	0.067*	
C16	−0.1353 (8)	0.3302 (10)	0.8718 (3)	0.057 (3)	
H16	−0.2108	0.3514	0.8568	0.069*	
C17	−0.1189 (7)	0.2206 (10)	0.8921 (3)	0.054 (2)	
H17	−0.1837	0.1665	0.8907	0.065*	
C18	−0.0078 (6)	0.1867 (8)	0.9149 (2)	0.0396 (19)	
C19	0.0009 (8)	0.0668 (10)	0.9347 (3)	0.055 (2)	
H19	−0.0685	0.0198	0.933	0.066*	
C20A	0.093 (2)	−0.085 (2)	0.9774 (9)	0.068 (9)	0.537 (10)
H20A	0.0772	−0.0557	1.0005	0.081*	0.537 (10)
H20B	0.0284	−0.1418	0.9649	0.081*	0.537 (10)
C21A	0.2022 (14)	−0.1538 (18)	0.9860 (6)	0.064 (5)	0.537 (10)
H21A	0.2672	−0.0959	0.9974	0.077*	0.537 (10)
H21B	0.2159	−0.1868	0.963	0.077*	0.537 (10)
C22A	0.202 (2)	−0.261 (3)	1.0117 (9)	0.082 (8)	0.537 (10)
C23A	0.111 (3)	−0.343 (3)	1.0118 (9)	0.086 (9)	0.537 (10)
H23A	0.0359	−0.3319	0.9953	0.103*	0.537 (10)
C24A	0.134 (2)	−0.444 (3)	1.0368 (7)	0.060 (6)	0.537 (10)
H24A	0.0799	−0.5052	1.0406	0.072*	0.537 (10)
C25A	0.251 (2)	−0.434 (2)	1.0547 (7)	0.056 (5)	0.537 (10)
H25A	0.2914	−0.4965	1.0713	0.067*	0.537 (10)
S1A	0.3179 (6)	−0.3043 (8)	1.0450 (2)	0.065 (2)	0.537 (10)
C20B	0.105 (3)	−0.108 (2)	0.9668 (9)	0.079 (12)	0.463 (10)
H20C	0.027	−0.1385	0.9674	0.095*	0.463 (10)
H20D	0.133	−0.162	0.9494	0.095*	0.463 (10)
C21B	0.185 (2)	−0.1203 (16)	1.0029 (5)	0.077 (7)	0.463 (10)
H21C	0.1558	−0.0674	1.0201	0.093*	0.463 (10)
H21D	0.2621	−0.0874	1.0022	0.093*	0.463 (10)
C22B	0.199 (3)	−0.251 (2)	1.0171 (10)	0.083 (9)	0.463 (10)
C23B	0.289 (3)	−0.300 (3)	1.0450 (9)	0.093 (13)	0.463 (10)
H23B	0.3579	−0.2514	1.0548	0.112*	0.463 (10)
C24B	0.279 (3)	−0.419 (3)	1.0585 (11)	0.083 (10)	0.463 (10)
H24B	0.3357	−0.4625	1.077	0.1*	0.463 (10)
C25B	0.170 (2)	−0.462 (3)	1.0394 (9)	0.069 (8)	0.463 (10)
H25B	0.1389	−0.5397	1.0447	0.083*	0.463 (10)
S1B	0.0946 (10)	−0.3640 (12)	1.0058 (4)	0.101 (5)	0.463 (10)
Au2	0.28143 (2)	0.48017 (3)	0.777307 (8)	0.03068 (8)	
Cl2	0.21265 (17)	0.32072 (19)	0.80665 (6)	0.0454 (5)	
N2	0.1363 (5)	0.6132 (6)	0.70443 (17)	0.0362 (15)	
P2	0.35877 (15)	0.64601 (18)	0.75586 (5)	0.0289 (4)	
C26	0.5016 (6)	0.6658 (7)	0.7892 (2)	0.0299 (16)	
C27	0.5040 (6)	0.6648 (7)	0.8265 (2)	0.0379 (18)	
H27	0.4327	0.6621	0.8334	0.046*	
C28	0.6086 (7)	0.6677 (8)	0.8532 (2)	0.044 (2)	
H28	0.6089	0.6705	0.8783	0.053*	
C29	0.7130 (7)	0.6664 (8)	0.8439 (2)	0.045 (2)	
H29	0.7851	0.6663	0.8625	0.054*	
C30	0.7121 (7)	0.6652 (8)	0.8075 (2)	0.046 (2)	
H30	0.7838	0.6636	0.8009	0.056*	
C31	0.6057 (6)	0.6661 (7)	0.7802 (2)	0.0356 (18)	
H31	0.6058	0.667	0.7551	0.043*	
C32	0.3900 (6)	0.6424 (7)	0.7114 (2)	0.0300 (16)	
C33	0.3777 (6)	0.5337 (8)	0.6909 (2)	0.0378 (18)	
H33	0.3527	0.4583	0.6999	0.045*	
C34	0.4022 (7)	0.5348 (9)	0.6566 (2)	0.045 (2)	
H34	0.3914	0.4609	0.6419	0.054*	
C35	0.4417 (7)	0.6435 (9)	0.6446 (2)	0.045 (2)	
H35	0.4592	0.644	0.6215	0.054*	
C36	0.4565 (7)	0.7507 (9)	0.6650 (2)	0.046 (2)	
H36	0.4849	0.8248	0.6563	0.056*	
C37	0.4300 (6)	0.7518 (8)	0.6987 (2)	0.0366 (18)	
H37	0.4391	0.8268	0.7129	0.044*	
C38	0.2807 (6)	0.7954 (7)	0.75625 (19)	0.0303 (16)	
C39	0.3250 (7)	0.8907 (6)	0.77927 (19)	0.0331 (17)	
H39	0.3999	0.876	0.7957	0.04*	
C40	0.2783 (7)	1.0025 (7)	0.7822 (2)	0.0392 (19)	
H40	0.3196	1.065	0.7986	0.047*	
C41	0.1638 (7)	1.0238 (8)	0.7596 (2)	0.044 (2)	
H41	0.1243	1.1007	0.7608	0.052*	
C42	0.1113 (7)	0.9300 (7)	0.7357 (2)	0.0401 (19)	
H42	0.0352	0.9443	0.72	0.048*	
C43	0.1660 (6)	0.8137 (7)	0.7336 (2)	0.0346 (17)	
C44	0.0976 (6)	0.7203 (8)	0.7085 (2)	0.0397 (19)	
H44	0.0205	0.7416	0.6948	0.048*	
C45	0.0589 (8)	0.5299 (8)	0.6788 (2)	0.050 (2)	
H45A	0.0829	0.5258	0.6557	0.06*	
H45B	−0.0221	0.5628	0.673	0.06*	
C46	0.0628 (8)	0.3992 (9)	0.6952 (3)	0.057 (2)	
H46A	0.1442	0.3675	0.701	0.069*	
H46B	0.0404	0.4048	0.7186	0.069*	
C47	−0.0150 (7)	0.3080 (8)	0.6709 (2)	0.0476 (18)	
C48A	−0.093 (3)	0.224 (3)	0.6782 (7)	0.054 (6)	0.299 (9)
H48A	−0.1008	0.2163	0.7025	0.065*	0.299 (9)
C49A	−0.161 (4)	0.150 (4)	0.6508 (9)	0.059 (8)	0.299 (9)
H49A	−0.2182	0.0908	0.6532	0.071*	0.299 (9)
C50A	−0.129 (4)	0.179 (4)	0.6195 (9)	0.060 (7)	0.299 (9)
H50A	−0.1598	0.1374	0.5966	0.072*	0.299 (9)
S2A	−0.0301 (12)	0.2967 (13)	0.6264 (3)	0.054 (3)	0.299 (9)
C48B	−0.0478 (19)	0.293 (2)	0.6343 (4)	0.062 (5)	0.701 (9)
H48B	−0.0151	0.3459	0.6194	0.075*	0.701 (9)
C49B	−0.128 (2)	0.2032 (18)	0.6183 (6)	0.073 (5)	0.701 (9)
H49B	−0.1556	0.1863	0.5927	0.088*	0.701 (9)
C50B	−0.160 (2)	0.142 (2)	0.6457 (4)	0.058 (4)	0.701 (9)
H50B	−0.2136	0.0738	0.6417	0.07*	0.701 (9)
S2B	−0.0933 (4)	0.2027 (4)	0.68793 (11)	0.0512 (13)	0.701 (9)

### Atomic displacement parameters (Å^2^)

**Table:**

	*U*^11^	*U*^22^	*U*^33^	*U*^12^	*U*^13^	*U*^23^
Au1	0.03364 (16)	0.0476 (2)	0.02662 (15)	−0.00584 (13)	0.00630 (12)	−0.00317 (13)
Cl1	0.0577 (13)	0.0663 (15)	0.0277 (10)	−0.0160 (11)	0.0119 (9)	−0.0042 (10)
N1	0.048 (4)	0.069 (5)	0.052 (5)	−0.013 (4)	0.016 (4)	0.007 (4)
P1	0.0283 (9)	0.0448 (12)	0.0264 (10)	−0.0059 (8)	0.0049 (8)	−0.0024 (9)
C1	0.027 (4)	0.031 (4)	0.038 (4)	0.004 (3)	0.006 (3)	0.003 (3)
C2	0.051 (5)	0.053 (5)	0.037 (5)	−0.010 (4)	0.013 (4)	−0.006 (4)
C3	0.063 (6)	0.038 (5)	0.059 (6)	−0.011 (4)	0.021 (5)	−0.007 (4)
C4	0.047 (5)	0.039 (5)	0.071 (7)	−0.006 (4)	0.014 (5)	0.009 (5)
C5	0.050 (5)	0.046 (5)	0.054 (6)	0.004 (4)	0.019 (4)	0.012 (4)
C6	0.040 (4)	0.040 (5)	0.044 (5)	0.001 (4)	0.012 (4)	0.001 (4)
C7	0.029 (4)	0.040 (5)	0.041 (5)	−0.008 (3)	0.013 (3)	0.002 (4)
C8	0.044 (5)	0.051 (5)	0.038 (5)	0.007 (4)	0.011 (4)	0.007 (4)
C9	0.050 (5)	0.056 (6)	0.059 (6)	0.018 (5)	0.020 (5)	0.022 (5)
C10	0.078 (7)	0.039 (5)	0.065 (7)	0.001 (5)	0.043 (6)	0.006 (5)
C11	0.069 (6)	0.053 (6)	0.051 (6)	−0.014 (5)	0.024 (5)	−0.015 (5)
C12	0.038 (4)	0.053 (5)	0.037 (5)	0.001 (4)	0.009 (4)	−0.006 (4)
C13	0.029 (4)	0.053 (5)	0.028 (4)	−0.001 (4)	0.005 (3)	−0.008 (4)
C14	0.037 (4)	0.065 (6)	0.042 (5)	0.003 (4)	0.004 (4)	0.009 (4)
C15	0.043 (5)	0.070 (7)	0.049 (6)	0.009 (5)	0.003 (4)	0.007 (5)
C16	0.034 (5)	0.074 (7)	0.058 (6)	0.006 (5)	0.004 (4)	−0.006 (5)
C17	0.030 (4)	0.073 (7)	0.056 (6)	−0.010 (4)	0.008 (4)	−0.029 (5)
C18	0.034 (4)	0.048 (5)	0.037 (5)	−0.002 (4)	0.010 (3)	−0.013 (4)
C19	0.044 (5)	0.068 (7)	0.055 (6)	−0.023 (5)	0.017 (4)	−0.014 (5)
C20A	0.056 (12)	0.083 (17)	0.070 (19)	0.007 (10)	0.028 (12)	0.035 (13)
C21A	0.051 (9)	0.066 (12)	0.059 (12)	−0.008 (8)	−0.012 (9)	0.002 (8)
C22A	0.066 (12)	0.083 (15)	0.081 (16)	0.001 (9)	−0.006 (11)	0.026 (12)
C23A	0.050 (11)	0.09 (2)	0.11 (2)	0.015 (11)	0.008 (12)	0.035 (14)
C24A	0.056 (12)	0.073 (13)	0.057 (13)	0.010 (10)	0.024 (10)	−0.004 (9)
C25A	0.053 (11)	0.080 (12)	0.042 (11)	0.022 (8)	0.026 (8)	0.008 (9)
S1A	0.049 (3)	0.090 (5)	0.054 (4)	0.007 (3)	0.013 (2)	0.002 (3)
C20B	0.09 (2)	0.084 (18)	0.057 (17)	−0.052 (16)	0.014 (14)	0.012 (13)
C21B	0.124 (19)	0.046 (9)	0.055 (13)	−0.012 (11)	0.013 (13)	−0.030 (9)
C22B	0.094 (17)	0.054 (11)	0.081 (17)	−0.030 (11)	−0.013 (13)	−0.011 (11)
C23B	0.072 (18)	0.076 (18)	0.12 (2)	−0.010 (13)	−0.002 (15)	0.000 (15)
C24B	0.066 (15)	0.095 (18)	0.09 (2)	−0.006 (14)	0.018 (11)	0.015 (15)
C25B	0.051 (15)	0.065 (14)	0.098 (17)	0.012 (11)	0.032 (12)	0.010 (12)
S1B	0.063 (6)	0.082 (6)	0.137 (9)	−0.025 (5)	−0.006 (5)	0.038 (6)
Au2	0.02441 (13)	0.02998 (15)	0.03575 (16)	−0.00214 (12)	0.00485 (11)	0.00045 (13)
Cl2	0.0407 (11)	0.0350 (11)	0.0600 (13)	−0.0067 (8)	0.0129 (9)	0.0058 (9)
N2	0.031 (3)	0.036 (4)	0.038 (4)	0.002 (3)	0.004 (3)	−0.005 (3)
P2	0.0231 (9)	0.0310 (11)	0.0320 (10)	−0.0026 (7)	0.0064 (7)	−0.0006 (8)
C26	0.026 (3)	0.025 (4)	0.036 (4)	−0.003 (3)	0.003 (3)	−0.003 (3)
C27	0.033 (4)	0.040 (5)	0.040 (5)	−0.007 (3)	0.009 (3)	0.000 (4)
C28	0.045 (5)	0.039 (5)	0.040 (5)	−0.008 (4)	−0.001 (4)	−0.001 (4)
C29	0.032 (4)	0.049 (5)	0.045 (5)	0.007 (4)	−0.005 (4)	−0.008 (4)
C30	0.032 (4)	0.054 (6)	0.050 (5)	0.002 (4)	0.007 (4)	−0.008 (4)
C31	0.031 (4)	0.039 (5)	0.035 (4)	−0.001 (3)	0.008 (3)	−0.007 (3)
C32	0.022 (3)	0.031 (4)	0.034 (4)	0.003 (3)	0.004 (3)	0.003 (3)
C33	0.030 (4)	0.039 (5)	0.041 (5)	0.007 (3)	0.003 (3)	−0.002 (4)
C34	0.041 (4)	0.055 (6)	0.039 (5)	0.017 (4)	0.007 (4)	−0.011 (4)
C35	0.038 (4)	0.058 (6)	0.042 (5)	0.014 (4)	0.014 (4)	0.005 (4)
C36	0.042 (5)	0.057 (6)	0.042 (5)	0.004 (4)	0.014 (4)	0.013 (4)
C37	0.034 (4)	0.040 (5)	0.036 (4)	0.004 (3)	0.008 (3)	0.001 (3)
C38	0.030 (4)	0.034 (4)	0.028 (4)	0.000 (3)	0.012 (3)	0.000 (3)
C39	0.073 (5)	0.008 (3)	0.021 (4)	−0.009 (3)	0.018 (4)	0.001 (3)
C40	0.043 (4)	0.036 (5)	0.039 (5)	−0.008 (4)	0.013 (4)	−0.003 (4)
C41	0.044 (5)	0.045 (5)	0.048 (5)	0.007 (4)	0.024 (4)	0.006 (4)
C42	0.037 (4)	0.033 (4)	0.048 (5)	0.006 (3)	0.006 (4)	0.002 (4)
C43	0.032 (4)	0.030 (4)	0.042 (5)	−0.004 (3)	0.009 (3)	0.003 (3)
C44	0.024 (4)	0.043 (5)	0.046 (5)	−0.001 (3)	−0.003 (3)	0.011 (4)
C45	0.050 (5)	0.042 (5)	0.046 (5)	−0.006 (4)	−0.008 (4)	0.007 (4)
C46	0.047 (5)	0.057 (6)	0.065 (6)	−0.009 (4)	0.009 (5)	−0.005 (5)
C47	0.041 (4)	0.040 (4)	0.058 (4)	−0.001 (3)	0.007 (3)	−0.003 (3)
C48A	0.048 (13)	0.040 (13)	0.072 (9)	−0.001 (9)	0.014 (10)	0.000 (10)
C49A	0.056 (15)	0.032 (15)	0.081 (14)	−0.004 (10)	0.003 (11)	0.007 (12)
C50A	0.071 (14)	0.028 (13)	0.068 (10)	−0.004 (9)	−0.004 (11)	0.001 (10)
S2A	0.059 (6)	0.041 (6)	0.057 (4)	−0.007 (4)	0.005 (4)	−0.003 (4)
C48B	0.084 (11)	0.042 (9)	0.057 (5)	−0.014 (7)	0.011 (7)	0.003 (7)
C49B	0.109 (11)	0.041 (10)	0.056 (4)	−0.015 (8)	0.001 (7)	−0.006 (6)
C50B	0.061 (10)	0.043 (9)	0.072 (6)	−0.012 (6)	0.021 (7)	−0.021 (7)
S2B	0.052 (2)	0.046 (2)	0.061 (2)	−0.0130 (17)	0.0245 (18)	−0.0151 (18)

### Geometric parameters (Å, °)

**Table:**

Au1—P1	2.235 (2)	C24B—H24B	0.95
Au1—Cl1	2.286 (2)	C25B—S1B	1.695 (17)
N1—C19	1.254 (11)	C25B—H25B	0.95
N1—C20A	1.471 (18)	Au2—P2	2.237 (2)
N1—C20B	1.484 (19)	Au2—Cl2	2.292 (2)
P1—C7	1.826 (8)	N2—C44	1.252 (10)
P1—C1	1.828 (8)	N2—C45	1.444 (10)
P1—C13	1.835 (7)	P2—C32	1.818 (8)
C1—C2	1.377 (11)	P2—C26	1.832 (7)
C1—C6	1.403 (11)	P2—C38	1.840 (7)
C2—C3	1.395 (12)	C26—C31	1.368 (10)
C2—H2	0.95	C26—C27	1.403 (10)
C3—C4	1.350 (12)	C27—C28	1.371 (10)
C3—H3	0.95	C27—H27	0.95
C4—C5	1.404 (13)	C28—C29	1.375 (12)
C4—H4	0.95	C28—H28	0.95
C5—C6	1.353 (11)	C29—C30	1.371 (12)
C5—H5	0.95	C29—H29	0.95
C6—H6	0.95	C30—C31	1.400 (10)
C7—C12	1.366 (11)	C30—H30	0.95
C7—C8	1.380 (11)	C31—H31	0.95
C8—C9	1.397 (12)	C32—C33	1.376 (10)
C8—H8	0.95	C32—C37	1.389 (10)
C9—C10	1.377 (13)	C33—C34	1.401 (11)
C9—H9	0.95	C33—H33	0.95
C10—C11	1.359 (13)	C34—C35	1.370 (12)
C10—H10	0.95	C34—H34	0.95
C11—C12	1.383 (12)	C35—C36	1.361 (12)
C11—H11	0.95	C35—H35	0.95
C12—H12	0.95	C36—C37	1.392 (11)
C13—C14	1.398 (12)	C36—H36	0.95
C13—C18	1.403 (11)	C37—H37	0.95
C14—C15	1.400 (11)	C38—C39	1.346 (10)
C14—H14	0.95	C38—C43	1.413 (10)
C15—C16	1.359 (13)	C39—C40	1.327 (10)
C15—H15	0.95	C39—H39	0.95
C16—C17	1.379 (13)	C40—C41	1.415 (11)
C16—H16	0.95	C40—H40	0.95
C17—C18	1.413 (11)	C41—C42	1.376 (11)
C17—H17	0.95	C41—H41	0.95
C18—C19	1.466 (13)	C42—C43	1.407 (10)
C19—H19	0.95	C42—H42	0.95
C20A—C21A	1.45 (2)	C43—C44	1.458 (11)
C20A—H20A	0.99	C44—H44	0.95
C20A—H20B	0.99	C45—C46	1.516 (12)
C21A—C22A	1.50 (2)	C45—H45A	0.99
C21A—H21A	0.99	C45—H45B	0.99
C21A—H21B	0.99	C46—C47	1.476 (11)
C22A—C23A	1.383 (16)	C46—H46A	0.99
C22A—S1A	1.661 (14)	C46—H46B	0.99
C23A—C24A	1.405 (17)	C47—C48B	1.341 (15)
C23A—H23A	0.95	C47—C48A	1.371 (18)
C24A—C25A	1.378 (15)	C47—S2A	1.646 (12)
C24A—H24A	0.95	C47—S2B	1.689 (9)
C25A—S1A	1.680 (16)	C48A—C49A	1.370 (13)
C25A—H25A	0.95	C48A—H48A	0.95
C20B—C21B	1.44 (2)	C49A—C50A	1.369 (13)
C20B—H20C	0.99	C49A—H49A	0.95
C20B—H20D	0.99	C50A—S2A	1.692 (17)
C21B—C22B	1.49 (2)	C50A—H50A	0.95
C21B—H21C	0.99	C48B—C49B	1.369 (12)
C21B—H21D	0.99	C48B—H48B	0.95
C22B—C23B	1.382 (16)	C49B—C50B	1.362 (11)
C22B—S1B	1.695 (16)	C49B—H49B	0.95
C23B—C24B	1.383 (17)	C50B—S2B	1.706 (12)
C23B—H23B	0.95	C50B—H50B	0.95
C24B—C25B	1.381 (17)		
			
P1—Au1—Cl1	177.39 (8)	C23B—C24B—H24B	127.2
C19—N1—C20A	117.8 (12)	C24B—C25B—S1B	115 (3)
C19—N1—C20B	120.6 (13)	C24B—C25B—H25B	122.5
C7—P1—C1	102.6 (3)	S1B—C25B—H25B	122.5
C7—P1—C13	108.4 (4)	C25B—S1B—C22B	92.4 (13)
C1—P1—C13	104.4 (4)	P2—Au2—Cl2	172.63 (7)
C7—P1—Au1	114.2 (3)	C44—N2—C45	116.9 (7)
C1—P1—Au1	111.5 (3)	C32—P2—C26	104.9 (3)
C13—P1—Au1	114.6 (3)	C32—P2—C38	104.7 (3)
C2—C1—C6	118.3 (7)	C26—P2—C38	105.7 (3)
C2—C1—P1	120.5 (6)	C32—P2—Au2	121.6 (3)
C6—C1—P1	121.2 (6)	C26—P2—Au2	103.6 (2)
C1—C2—C3	120.8 (8)	C38—P2—Au2	114.8 (2)
C1—C2—H2	119.6	C31—C26—C27	118.5 (7)
C3—C2—H2	119.6	C31—C26—P2	123.9 (6)
C4—C3—C2	120.2 (9)	C27—C26—P2	117.1 (5)
C4—C3—H3	119.9	C28—C27—C26	120.5 (7)
C2—C3—H3	119.9	C28—C27—H27	119.8
C3—C4—C5	119.6 (8)	C26—C27—H27	119.8
C3—C4—H4	120.2	C27—C28—C29	120.8 (8)
C5—C4—H4	120.2	C27—C28—H28	119.6
C6—C5—C4	120.5 (8)	C29—C28—H28	119.6
C6—C5—H5	119.7	C30—C29—C28	119.4 (7)
C4—C5—H5	119.7	C30—C29—H29	120.3
C5—C6—C1	120.6 (8)	C28—C29—H29	120.3
C5—C6—H6	119.7	C29—C30—C31	120.2 (8)
C1—C6—H6	119.7	C29—C30—H30	119.9
C12—C7—C8	119.1 (8)	C31—C30—H30	119.9
C12—C7—P1	123.1 (6)	C26—C31—C30	120.6 (7)
C8—C7—P1	117.8 (6)	C26—C31—H31	119.7
C7—C8—C9	119.5 (8)	C30—C31—H31	119.7
C7—C8—H8	120.2	C33—C32—C37	120.1 (7)
C9—C8—H8	120.2	C33—C32—P2	121.5 (6)
C10—C9—C8	120.1 (8)	C37—C32—P2	118.4 (6)
C10—C9—H9	120	C32—C33—C34	119.8 (8)
C8—C9—H9	120	C32—C33—H33	120.1
C11—C10—C9	120.2 (9)	C34—C33—H33	120.1
C11—C10—H10	119.9	C35—C34—C33	119.3 (8)
C9—C10—H10	119.9	C35—C34—H34	120.4
C10—C11—C12	119.5 (9)	C33—C34—H34	120.4
C10—C11—H11	120.2	C36—C35—C34	121.3 (8)
C12—C11—H11	120.2	C36—C35—H35	119.3
C7—C12—C11	121.5 (8)	C34—C35—H35	119.3
C7—C12—H12	119.2	C35—C36—C37	120.0 (8)
C11—C12—H12	119.2	C35—C36—H36	120
C14—C13—C18	118.3 (7)	C37—C36—H36	120
C14—C13—P1	117.9 (6)	C32—C37—C36	119.4 (8)
C18—C13—P1	123.5 (6)	C32—C37—H37	120.3
C13—C14—C15	121.3 (9)	C36—C37—H37	120.3
C13—C14—H14	119.3	C39—C38—C43	115.7 (7)
C15—C14—H14	119.3	C39—C38—P2	123.0 (6)
C16—C15—C14	120.3 (9)	C43—C38—P2	121.3 (6)
C16—C15—H15	119.8	C40—C39—C38	128.8 (8)
C14—C15—H15	119.8	C40—C39—H39	115.6
C15—C16—C17	119.6 (8)	C38—C39—H39	115.6
C15—C16—H16	120.2	C39—C40—C41	116.6 (8)
C17—C16—H16	120.2	C39—C40—H40	121.7
C16—C17—C18	121.6 (8)	C41—C40—H40	121.7
C16—C17—H17	119.2	C42—C41—C40	118.1 (8)
C18—C17—H17	119.2	C42—C41—H41	120.9
C13—C18—C17	118.8 (8)	C40—C41—H41	120.9
C13—C18—C19	123.8 (7)	C41—C42—C43	122.5 (7)
C17—C18—C19	117.3 (8)	C41—C42—H42	118.7
N1—C19—C18	122.5 (8)	C43—C42—H42	118.7
N1—C19—H19	118.7	C42—C43—C38	118.1 (7)
C18—C19—H19	118.7	C42—C43—C44	116.7 (7)
C21A—C20A—N1	111.3 (18)	C38—C43—C44	125.2 (7)
C21A—C20A—H20A	109.4	N2—C44—C43	122.7 (7)
N1—C20A—H20A	109.4	N2—C44—H44	118.6
C21A—C20A—H20B	109.4	C43—C44—H44	118.6
N1—C20A—H20B	109.4	N2—C45—C46	110.1 (7)
H20A—C20A—H20B	108	N2—C45—H45A	109.6
C20A—C21A—C22A	111.7 (17)	C46—C45—H45A	109.6
C20A—C21A—H21A	109.3	N2—C45—H45B	109.6
C22A—C21A—H21A	109.3	C46—C45—H45B	109.6
C20A—C21A—H21B	109.3	H45A—C45—H45B	108.2
C22A—C21A—H21B	109.3	C47—C46—C45	113.7 (8)
H21A—C21A—H21B	107.9	C47—C46—H46A	108.8
C23A—C22A—C21A	128.2 (17)	C45—C46—H46A	108.8
C23A—C22A—S1A	108.2 (15)	C47—C46—H46B	108.8
C21A—C22A—S1A	123.6 (15)	C45—C46—H46B	108.8
C22A—C23A—C24A	118 (3)	H46A—C46—H46B	107.7
C22A—C23A—H23A	120.9	C48B—C47—C48A	96.2 (18)
C24A—C23A—H23A	120.9	C48B—C47—C46	132.7 (11)
C25A—C24A—C23A	105 (3)	C48A—C47—C46	130.3 (13)
C25A—C24A—H24A	127.6	C48A—C47—S2A	105.4 (12)
C23A—C24A—H24A	127.6	C46—C47—S2A	124.2 (7)
C24A—C25A—S1A	115 (2)	C48B—C47—S2B	106.2 (10)
C24A—C25A—H25A	122.3	C46—C47—S2B	120.9 (7)
S1A—C25A—H25A	122.3	S2A—C47—S2B	114.9 (6)
C22A—S1A—C25A	92.7 (11)	C49A—C48A—C47	121 (2)
C21B—C20B—N1	111.7 (19)	C49A—C48A—H48A	119.7
C21B—C20B—H20C	109.3	C47—C48A—H48A	119.7
N1—C20B—H20C	109.3	C50A—C49A—C48A	107 (2)
C21B—C20B—H20D	109.3	C50A—C49A—H49A	126.6
N1—C20B—H20D	109.3	C48A—C49A—H49A	126.6
H20C—C20B—H20D	107.9	C49A—C50A—S2A	112 (2)
C20B—C21B—C22B	114.1 (16)	C49A—C50A—H50A	124.2
C20B—C21B—H21C	108.7	S2A—C50A—H50A	124.2
C22B—C21B—H21C	108.7	C47—S2A—C50A	95.3 (12)
C20B—C21B—H21D	108.7	C47—C48B—C49B	120.9 (19)
C22B—C21B—H21D	108.7	C47—C48B—H48B	119.6
H21C—C21B—H21D	107.6	C49B—C48B—H48B	119.6
C23B—C22B—C21B	128.1 (19)	C50B—C49B—C48B	107 (2)
C23B—C22B—S1B	106.9 (14)	C50B—C49B—H49B	126.3
C21B—C22B—S1B	124.4 (16)	C48B—C49B—H49B	126.3
C22B—C23B—C24B	120 (3)	C49B—C50B—S2B	112.2 (15)
C22B—C23B—H23B	120.1	C49B—C50B—H50B	123.9
C24B—C23B—H23B	120.1	S2B—C50B—H50B	123.9
C25B—C24B—C23B	106 (3)	C47—S2B—C50B	93.2 (8)
C25B—C24B—H24B	127.2		
			
C7—P1—C1—C2	−144.9 (6)	Au2—P2—C26—C31	123.7 (6)
C13—P1—C1—C2	102.0 (7)	C32—P2—C26—C27	−176.6 (6)
Au1—P1—C1—C2	−22.3 (7)	C38—P2—C26—C27	73.0 (6)
C7—P1—C1—C6	33.5 (7)	Au2—P2—C26—C27	−48.1 (6)
C13—P1—C1—C6	−79.6 (7)	C31—C26—C27—C28	1.8 (11)
Au1—P1—C1—C6	156.2 (5)	P2—C26—C27—C28	174.1 (6)
C6—C1—C2—C3	0.1 (12)	C26—C27—C28—C29	−2.7 (12)
P1—C1—C2—C3	178.6 (7)	C27—C28—C29—C30	1.5 (13)
C1—C2—C3—C4	−0.6 (14)	C28—C29—C30—C31	0.5 (13)
C2—C3—C4—C5	1.3 (14)	C27—C26—C31—C30	0.2 (11)
C3—C4—C5—C6	−1.6 (13)	P2—C26—C31—C30	−171.6 (6)
C4—C5—C6—C1	1.2 (12)	C29—C30—C31—C26	−1.3 (13)
C2—C1—C6—C5	−0.4 (11)	C26—P2—C32—C33	110.4 (6)
P1—C1—C6—C5	−178.9 (6)	C38—P2—C32—C33	−138.5 (6)
C1—P1—C7—C12	−100.5 (7)	Au2—P2—C32—C33	−6.3 (7)
C13—P1—C7—C12	9.5 (8)	C26—P2—C32—C37	−67.9 (6)
Au1—P1—C7—C12	138.7 (6)	C38—P2—C32—C37	43.1 (6)
C1—P1—C7—C8	76.3 (7)	Au2—P2—C32—C37	175.4 (4)
C13—P1—C7—C8	−173.7 (6)	C37—C32—C33—C34	−2.0 (10)
Au1—P1—C7—C8	−44.5 (7)	P2—C32—C33—C34	179.7 (5)
C12—C7—C8—C9	−0.9 (12)	C32—C33—C34—C35	2.1 (11)
P1—C7—C8—C9	−177.8 (6)	C33—C34—C35—C36	−0.7 (12)
C7—C8—C9—C10	−0.9 (13)	C34—C35—C36—C37	−0.8 (12)
C8—C9—C10—C11	0.8 (14)	C33—C32—C37—C36	0.5 (11)
C9—C10—C11—C12	0.9 (14)	P2—C32—C37—C36	178.9 (6)
C8—C7—C12—C11	2.7 (13)	C35—C36—C37—C32	0.9 (11)
P1—C7—C12—C11	179.4 (7)	C32—P2—C38—C39	−114.3 (6)
C10—C11—C12—C7	−2.7 (14)	C26—P2—C38—C39	−3.8 (7)
C7—P1—C13—C14	−111.2 (7)	Au2—P2—C38—C39	109.7 (6)
C1—P1—C13—C14	−2.4 (7)	C32—P2—C38—C43	69.7 (6)
Au1—P1—C13—C14	119.9 (6)	C26—P2—C38—C43	−179.7 (6)
C7—P1—C13—C18	76.3 (7)	Au2—P2—C38—C43	−66.3 (6)
C1—P1—C13—C18	−174.9 (6)	C43—C38—C39—C40	−4.2 (12)
Au1—P1—C13—C18	−52.7 (7)	P2—C38—C39—C40	179.6 (6)
C18—C13—C14—C15	−2.0 (13)	C38—C39—C40—C41	3.7 (12)
P1—C13—C14—C15	−175.0 (7)	C39—C40—C41—C42	−2.1 (11)
C13—C14—C15—C16	1.5 (14)	C40—C41—C42—C43	1.5 (12)
C14—C15—C16—C17	−0.7 (14)	C41—C42—C43—C38	−2.1 (12)
C15—C16—C17—C18	0.4 (14)	C41—C42—C43—C44	176.6 (8)
C14—C13—C18—C17	1.7 (11)	C39—C38—C43—C42	3.1 (10)
P1—C13—C18—C17	174.2 (6)	P2—C38—C43—C42	179.3 (6)
C14—C13—C18—C19	179.2 (8)	C39—C38—C43—C44	−175.5 (7)
P1—C13—C18—C19	−8.3 (11)	P2—C38—C43—C44	0.7 (11)
C16—C17—C18—C13	−1.0 (12)	C45—N2—C44—C43	179.7 (7)
C16—C17—C18—C19	−178.6 (8)	C42—C43—C44—N2	−179.6 (8)
C20A—N1—C19—C18	169.5 (18)	C38—C43—C44—N2	−1.0 (13)
C20B—N1—C19—C18	−167 (2)	C44—N2—C45—C46	−135.6 (8)
C13—C18—C19—N1	−2.3 (14)	N2—C45—C46—C47	179.4 (7)
C17—C18—C19—N1	175.2 (9)	C45—C46—C47—C48B	34.4 (18)
C19—N1—C20A—C21A	157 (2)	C45—C46—C47—C48A	−133 (2)
C20B—N1—C20A—C21A	54 (4)	C45—C46—C47—S2A	42.5 (14)
N1—C20A—C21A—C22A	177 (2)	C45—C46—C47—S2B	−139.4 (8)
C20A—C21A—C22A—C23A	40 (4)	C48B—C47—C48A—C49A	7(2)
C20A—C21A—C22A—S1A	−143 (3)	C46—C47—C48A—C49A	177 (2)
C21A—C22A—C23A—C24A	175 (4)	S2A—C47—C48A—C49A	1.7 (19)
S1A—C22A—C23A—C24A	−2.6 (18)	S2B—C47—C48A—C49A	−150 (10)
C22A—C23A—C24A—C25A	−2.7 (18)	C47—C48A—C49A—C50A	1(2)
C23A—C24A—C25A—S1A	7(2)	C48A—C49A—C50A—S2A	−4(3)
C23A—C22A—S1A—C25A	5.5 (18)	C48B—C47—S2A—C50A	−33 (8)
C21A—C22A—S1A—C25A	−172 (3)	C48A—C47—S2A—C50A	−3(2)
C24A—C25A—S1A—C22A	−8(2)	C46—C47—S2A—C50A	−179 (2)
C19—N1—C20B—C21B	−144 (2)	S2B—C47—S2A—C50A	2(2)
C20A—N1—C20B—C21B	−56 (4)	C49A—C50A—S2A—C47	4(3)
N1—C20B—C21B—C22B	−179 (3)	C48A—C47—C48B—C49B	−6(3)
C20B—C21B—C22B—C23B	161 (4)	C46—C47—C48B—C49B	−176.3 (14)
C20B—C21B—C22B—S1B	−30 (5)	S2A—C47—C48B—C49B	145 (8)
C21B—C22B—C23B—C24B	170 (5)	S2B—C47—C48B—C49B	−1.8 (16)
S1B—C22B—C23B—C24B	−1.6 (18)	C47—C48B—C49B—C50B	0.2 (19)
C22B—C23B—C24B—C25B	−2(2)	C48B—C49B—C50B—S2B	1.6 (19)
C23B—C24B—C25B—S1B	4(3)	C48B—C47—S2B—C50B	2.2 (14)
C24B—C25B—S1B—C22B	−5(3)	C48A—C47—S2B—C50B	26 (10)
C23B—C22B—S1B—C25B	3(2)	C46—C47—S2B—C50B	177.5 (11)
C21B—C22B—S1B—C25B	−168 (4)	S2A—C47—S2B—C50B	−4.2 (12)
C32—P2—C26—C31	−4.8 (7)	C49B—C50B—S2B—C47	−2.3 (17)
C38—P2—C26—C31	−115.2 (7)		

### Hydrogen-bond geometry (Å, °)

**Table:**

*D*—H···*A*	*D*—H	H···*A*	*D*···*A*	*D*—H···*A*
C28—H28···Cl1^i^	0.95	2.81	3.454 (9)	126

Symmetry codes: (i) −*x*+1, −*y*+1, −*z*+2.
